# Design to Assist Better Youthhood for Adolescents with Lower-Limb Disability through Virtual Reality Sports

**DOI:** 10.3390/ijerph19073985

**Published:** 2022-03-27

**Authors:** Xiaochen Zhang, Lanxin Hui, Muge Li, Jiajing Huang, Chengyuan Chen, Yunping Yang, Fuchuan Song, Fei Hu, Ding-Bang Luh

**Affiliations:** 1 Department of Industrial Design, Guangdong University of Technology, Guangzhou 510090, China; xzhang@gdut.edu.cn (X.Z.); 2111917097@mail2.gdut.edu.cn (L.H.); 2112017090@mail2.gdut.edu.cn (M.L.); 2111917071@mail2.gdut.edu.cn (J.H.); 2112017010@mail2.gdut.edu.cn (C.C.); 2111917032@mail2.gdut.edu.cn (Y.Y.); 2 F11X Silicon Photonics Process Development, Intel Corporation, Albuquerque, NM 87124, USA; fuchuan.song@intel.com; 3 College of Design and Innovation, Tongji University, Shanghai 200082, China; hufei@tongji.edu.cn

**Keywords:** flow, lower-limb disability, rehabilitation, rock-climbing, virtual reality sports

## Abstract

Background: Youths with lower limb dysfunction display low levels of sports participation, which limits their growth and self-development, both physically and mentally. Recently, VR technology has proven its profound value in the psychological evaluation and treatment, rehabilitation, and immersive training of people in need. We have proposed, designed, and developed a VR rock-climbing game for youths with lower-limb dysfunction that allows them to engage in enjoyable and purposeful in-game tasks that simultaneously bring about intensive real-world exercise. Methods: Pilot studies were conducted on college students whose lower limbs were fixed to chairs. Heart rate monitoring, a flow questionnaire, interviews, and observation were conducted for each participant to evaluate the impact of the VR rock-climbing game. The collected data were trimmed on the basis of Cronbach’s alpha and corrected item–total correlation (CITC) to guarantee the data’s reliability. Results: The average value of each flow experience dimension was greater than 4 (0.76 < SD < 0.91). According to the flow-based analysis and the whole-process feeling distribution (WPFD), the evaluated study brought about the participants’ happiness and a sense of mastery and achievement. Conclusions: By bringing about a deep and enjoyable immersion in VR, it remarkably promotes the participants’ intention to participate in exercises.

## 1. Introduction

According to the public report of the World Health Organization (WHO), in 2020, 15% of the world’s population suffered from disabilities [1,2]. Approximately 3.7% of Canadian children and 5.6% of American children have a disability [3]. For people who suffer from disabilities, sports are considered efficient tools within the framework of disability management and have positive effects that can improve their psycho-physical status [4]. According to the American College of Sports Medicine (ACSM) and the American Heart Association (AHA), individuals require moderate-intensity physical aerobic activity, while the Centers for Disease Control and Prevention recognize that individuals with physical disabilities have barriers that hinder physical activities [5]. It is widely agreed [6] that participating in sports is beneficial for psycho-social health for youth with disability since physical activity improves their fitness and overall functioning, improves their motor skills and functional activities, and promotes health and social integration [7]. Youths with disability engage in less physical activity compared with their typically developing peers, often resulting in delayed gross motor development, less proficiency in balance and coordination, poor cardiovascular fitness [8], and a loss of opportunity to promote inclusion, physical functioning, and overall well-being [7].

Low levels of physical activity are attributed to various functional deficits, especially those of the lower limbs, as these affect gait, mobility, and, consequently, physical activity [9]. Lower-limb disability may be caused, for example, by spinal cord injury, limb spasticity, or congenital aplasia. The lower-limb disabled who have limitations in ADL (activities of daily living), with great possibilities of serious falls [10], are not well motivated to participate in sports. With lower body esteem [11], they are less likely to exercise in public due to social fears. Additionally, maintaining regular physical activity and exercise in the home is challenging due to space restrictions.

In order to increase the opportunities for exercise and to develop enthusiasm to participate in sports and maintain long-term exercise habits, we have designed and developed VR rock-climbing software aimed at providing a simulated rock-climbing experience for youths with lower-limb dysfunction who may never experience real rock-climbing. By integrating moderate challenges and enjoyable task-oriented storylines in surreal scenes, the VR rock-climbing software introduced here is intended to allow youths with limb dysfunction to exercise and entertain themselves in the immersive scenario. 

To evaluate the software, we conducted a pilot study with adolescent participants whose lower bodies were fixed on swivel chairs and collected the flow interview statistics in a subsequent evaluation.

The contribution of this work is listed as follows.

1. A VR-based hybrid sports solution aimed at promoting willingness and efficiency in exercise participation, specifically designed for adolescents with lower-limb disability, is proposed and elaborately designed.
2. The designed VR solution was developed using Unity3D with HTC VIVE. In the scenes, novel design guidelines such as a semi-surreal style, wonder- and figure-based storylines, progressive challenges with instant gratification, and design-able action specifically designed for adolescents with lower-limb disability were introduced and applied.
3. Flow (psychology) was adapted in evaluating the designed and developed solution. Pilot studies with real-time field tests with adolescent college student volunteers were conducted. The video clip of the introduced solution can be viewed in the Supplemental files.

The rest of this paper is organized as follows: Section 2 reviews previous work presented in the literature. Section 3 introduces the method and materials. Section 4 presents the design of the experimental evaluation. Section 5 collects and analyzes the result following flow (psychology). Section 6 concludes the work.

## 2. Related Work

### 2.1. Virtual Reality for People with Disabilities

Weiss defines virtual reality as the use of interactive simulations created with computer hardware and software to present users with opportunities to engage in environments that appear and feel similar to real-world objects and events [12]. Mihelj believes VR comprises an interactive computer simulation that senses the user’s state and operation and replaces or augments sensory feedback information to one or more senses in such a way that the user has a sense of being immersed in the simulation (the virtual environment) [13]. Along with the conceptual evolution of VR in history, society has reached a consensus that there are three representative and essential features of VR, namely, immersion, presence, and interactivity [14].

In recent decades, VR technology has been widely applied in the fields of military training, psychotherapy, and the rehabilitation of the disabled, achieving impressive outcomes [15,16,17].

At the same time, virtual reality technology has proven to be of great value in motor rehabilitation, educational treatments, and psychological and communication interventions [18,19,20]. Postolache applied physical rehabilitation monitoring by combining serious VR games and the Wearable Sensor Network to improve patient engagement during physical rehabilitation [21]. VR-based rehabilitation training for youths with early-stage disabilities has preliminarily been verified experimentally. El-Shamy evaluated the effects of virtual reality solutions versus conventional physiotherapy on upper extremity function in youths with obstetric brachial plexus injury and found that the participants in the VR group showed better results in shoulder function post-treatment [22].

Moreover, VR-empowered exercise has been shown to have an observable positive impact on the recovery of balancing ability in youths with cerebral palsy (CP) [23,24]. By providing sports scenes in VR, these solutions provide a real-time interactive experience, with duplicated multi-channel senses for virtual training, but with low injury risks. Feelings of safety and entertainment have been widely reported for VR sports solutions that have been designed to break the barriers of attending sports for people with disability [25]. However, there have been limited efforts made to design VR solutions for youths with lower-limb disabilities.

### 2.2. Flow (Psychology) for VR

In order to better quantify the impact of VR sports games on the rehabilitation and mental health of youths with lower-limb dysfunction, we used the flow (psychology) theory to guide how the experiments were conducted and to evaluate the results. Flow is a state of conscious concentration so focused that it amounts to absolute absorption in an activity, making the experience genuinely satisfying [26]. There have been observations and empirical opinions that emphasize the importance of flow in VR [27]. The most commonly used methods to measure the flow experience are retrospective questionnaires and interviews. Researchers have also proposed evaluating the flow experience in virtual reality through physiological indicators [28,29].

At present, the most popular flow state scales that are generally recognized are the Flow State Scale (FSS) and the Dispositional Flow Scale (DFS) of Marsh and Jackson [30]. Furthermore, the dimensions of the flow experience scale questionnaire were mainly developed from the seven elements of flow experience proposed by Csikszentimihalyi [26,31]. In practice, scholars are always formulating customized flow tables according to the characteristics and needs of their particular research.

Among the influential studies, the flow scale studied by Hong et al. includes three flow dimensions (absorption, work enjoyment, and intrinsic work motivation) [32], while the flow chart studied by Lee et al. includes four flow dimensions (skills and challenges, focused attention, human and machine interactivity, and the performance of online game systems) [33]. Cowley et al. proposed a practical, integrated approach for analyzing the mechanics and esthetics of game-play using eight elements (a challenging but tractable task to complete, full immersion in the task, a feeling of full control, complete freedom to concentrate on the task, a task with clear and unambiguous goals, immediate feedback on actions, being less conscious of the passage of time, and a sense of identity that lessens but is reinforced afterward) to measure the flow experience in the game [34]. Bressler and Bodzin found that the three most popular flow factors in augmented reality games are concentration, the challenge–skill balance, and an intrinsically rewarding experience [35]. Faiola adapted virtual learning through telepresence in the context of game-based education [36].

The experimental questionnaire in this study is adapted from the flow experience scales used for VR game experiments. These include the GameFlow scale proposed by Sweetser, which covers concentration, challenge, player skills, control, clear goals, feedback, and social interaction [37], and the flow chart compiled by Hong et al. that uses three dimensions (a sense of control, enjoyment, and concentration) to measure the flow experience [38]. To better fit the context, we also referred to the study of Renard, which used a flow scale that includes both concentration on the activity and enjoyment derived from the activity [39], and the flow dimensions studied by Zhou, which includes three aspects: perceived enjoyment, concentration, and perceived control [40].

Our work combines the characteristics of VR games with reference to state-of-art flow studies. On the basis of the three important characteristics of VR (immersion, interaction, and existence), players have the opportunity to engage with an imaginary world to manipulate and interact with the environment through a blend of visual, sound, and haptic feedback. As a matter of fact, youths with lower-limb dysfunction prefer a stronger sense of accomplishment to achieve encouragement and attraction to be fully engaged. Thus, control, immersion, feedback, satisfaction, and accomplishment were taken as the primary flow experience dimensions in our study, as described below.

#### 2.2.1. Mastery

The sense of mastery (control, administrate) in a VR game refers to the user’s perception of the virtual environment and their own actions and operational levels during the process of participating in the VR game, including the matching between the tasks performed and their own operational capabilities as well as the clarity of the operational targets in the game. The operation skills of the controller and other devices in the VR game and the execution of the actions are required for the game to be performed coherently and smoothly. This is consistent with the concept of immersion and its measurement [38].

#### 2.2.2. Immersion

The sense of immersion in a VR environment includes full concentration, a sense of presence, and changes in the sense of time, which allow users to devote themselves to the virtual scene. This is consistent with the concept of immersion measured by Sweetser in the game flow model, which means deep participation within this context. In an ideal situation, users may ignore other perceivable information from the outside except those relevant to the designated tasks. One of the most intuitively understandable by-products is losing a sense of time. In the context of VR, presence is also a crucial subjective experience and is always coupled with immersion. Sometimes, they simply cause debate in distinguishing immersion and presence.

#### 2.2.3. Feedback

Timely feedback in VR means that users should receive appropriate and timely feedback. Moreover, the operations in the game should have timely and appropriate responses that provide timely operational feedback information to help players better experience the VR experience and complete the tasks. This is consistent with the concept of feedback, as measured by Raphael.

#### 2.2.4. Satisfaction

Satisfaction is defined as the sense of satisfaction that arises from the actual experience related to the expected experience. The construct of satisfaction used in this study is consistent with the concept of satisfaction in the study of Renard and Hsu et al. [39,41]. The measurement items include enjoyment and satisfaction in real-time, exploratory behavior after the field experience, as well as the willingness to communicate via personal and social channels.

#### 2.2.5. Accomplishment

**Figure 1:**
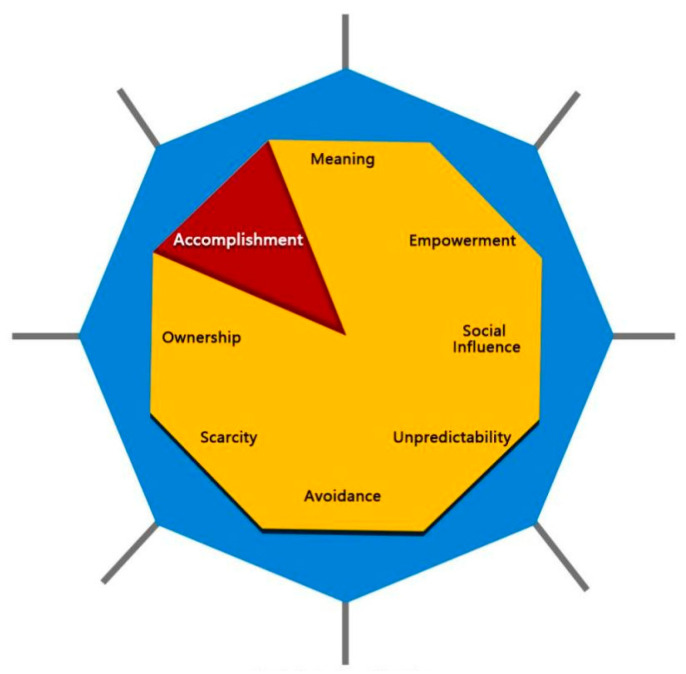
The octagonal behavior analysis graph.

Accomplishment is a dimension of the octagonal behavior analysis graph shown in Figure 1 [42]. In order to better collect feedback on the sports experience of youths with lower-limb dysfunction, we focused on progress and achievement in completing the tasks by setting the game levels progressively and planning to add rewards at the end of the game to encourage users to come back. In fact, overcoming difficulties in achieving rewards and experiencing a surreal experience are also meaningful forms of delivering a sense of accomplishment.

## 3. Method and Materials

In order to better evaluate the usefulness of virtual reality technology for young people with lower-limb disabilities, we integrated the sports game concept with the design of virtual reality solutions and realized a virtual reality design concept by developing a virtual reality application from scratch in the Unity with Head-Mounted Display (HMD) VR suite. In order to evaluate the developed VR sports software, we invited 25 youth volunteers to participate in the pilot study evaluation and applied the flow theory for collecting and analyzing the results.

### 3.1. Design Concept

In order to design VR software to help youths with lower-limb dysfunction improve their sports participation and body development, thus assisting them to achieve a better youthhood, we designed a surreal virtual scenario with a rock-climbing storyline. Games are naturally attractive to curious youths; in turn, this triggers the willingness to learn the rules and overcome the challenges through effort. Thus, we chose a rock-climbing game as the theme of the software. In its design, the game includes not only the perspective of entertainment but also psychological and physical perspectives. Psychologically, it enables youths with lower-limb dysfunction to experience sports in a similar to ordinary youths, making it possible for them to perceive the embodied experience and enjoy the freedom and happiness brought by specific exercise or activities. These experiences, gained through the virtual environment and multi-modal interaction, are of great significance to youths with lower-limb dysfunctions who have difficulty with mobility. Physiologically, immersive sports participation can bring about the enthusiasm of disabled youth to exercise, avoid the decline or even loss of athletic ability, and improve their physical health. In addition, compared with actual outdoor sports, virtual sports help to significantly reduce the possibility of accidental hazards causing injury. 

In fact, youths with lower-limb dysfunction pay a higher price for participating in various field sports. To break the limits of conventional sports, VR can be used as a basis for formulating a field of exercises for customized sports activities, allowing the users to embrace the missing sports opportunities and to strengthen their bodies.

### 3.2. Design Content

In order to enable youth with lower-limb dysfunction to gain courage, a sense of accomplishment, and learn exercise skills through a game, we proposed to develop a floating island as the setting, and we set up a storyline about rescuing islanders and integrated events and storylines from the real world. Historical knowledge related to famous buildings (such as the Leaning Tower of Pisa and the Colosseum) makes it both interesting and educational. The user has to use the HTC Vive VR suite to enter the virtual environment and then use the instantaneous movement and grab functions of the gamepad to complete the building-climbing tasks and win trophies. Specifically, the novelty of the introduced and developed VR rock-climbing has the following merits or highlights.

#### 3.2.1. Semi-Surreal Style

**Figure 2:**
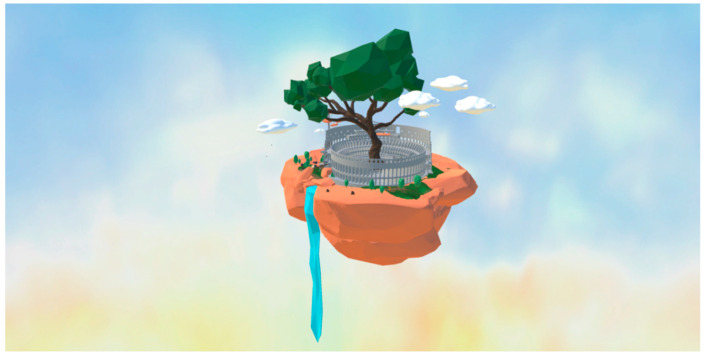
Low polygon-styled semi-surreal Roman Arena scene.

The low polygon style that forms the scene allows sufficient imagination in a fairy-tale-like world, while the illusion of color-matching also favors adolescents. In order to imitate real sports instead of being out of touch with reality, the scene elements and physical rules are restricted to the combinations and laws in the real world, as shown in Figure 2.

#### 3.2.2. Wonder- and Figure-Based Storyline

**Figure 3:**
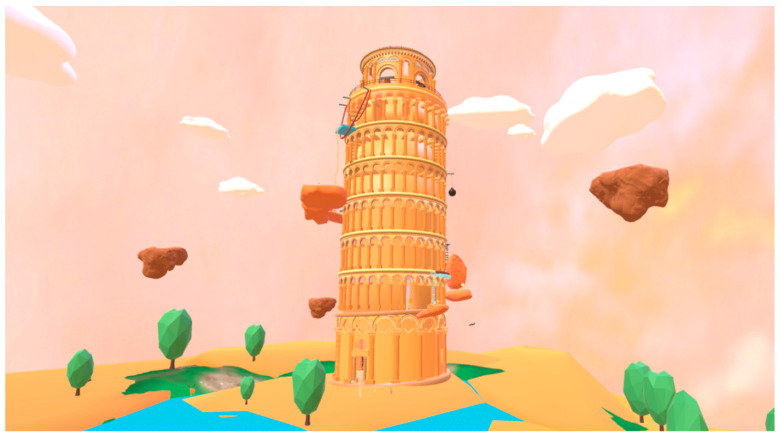
The climbing scene of the Leaning Tower of Pisa.

We made use of world-famous wonders such as the Leaning Tower of Pisa (Figure 3) and the Colosseum and historical figures such as Galileo and Spartan warriors to create meaningful and attractive game scenes and storylines so as to allow participants with lower-limb disabilities, who may have less chance for on-the-spot investigations, to visit the scenes and storylines while completing the tasks. Moreover, historical knowledge and rock-climbing-specific guides (such as the use of rock-climbing anti-skid powder and timely placing it when climbing to the top) also play a role, along with the progress.

#### 3.2.3. Progressive Challenges with Instant Gratification

**Figure 4:**
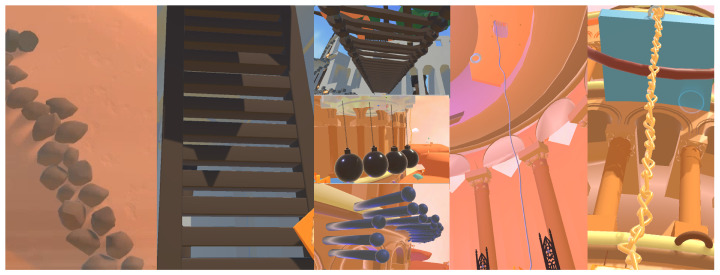
Climbing paths of rocks, ladders, iron ball paths, ropes, and iron chains.

In order to enable youths with lower-limb dysfunction to overcome their fears and temper their will by progressing through storyline-based challenges, the design follows the principle of moving from easy to difficult and from low to high as the game progresses. We set up obstacles such as moving gears to increase the difficulty of climbing so that the users can continuously improve their reaction and problem-solving skills in the process of coping with obstacles. Rich climbing paths, such as rocks, ladders, iron ball paths, ropes, and iron chains (Figure 4), with instant gratification rewards, create more willingness to play while reducing the fear of failure. Furthermore, different designs of climbing challenges enable participants to constantly think about climbing and grasping strategies, calculating body rotation angles, and choosing climbing paths during the activity so as to practice physically and mentally at the same time.

Rocks, iron, wood, ropes, iron chains, iron balls, and other climbing objects are set in the climbing scene. By simulating different sounds and vibration feedback for rocks, iron, wood, ropes, iron chains, and iron balls, the motion simulation is more realistic and attractive.

By bounding rigid-body components, we simulated the landing gravity when falling. A fear of falling is well simulated with pure visual cognition.

In terms of the interactive design, we introduced drone guidance and road signs to provide participants with route guidance and provided interactive feedback in the form of rock-climbing point discoloration and audio prompting.

#### 3.2.4. Design-Able Action on Purpose

**Figure 5:**
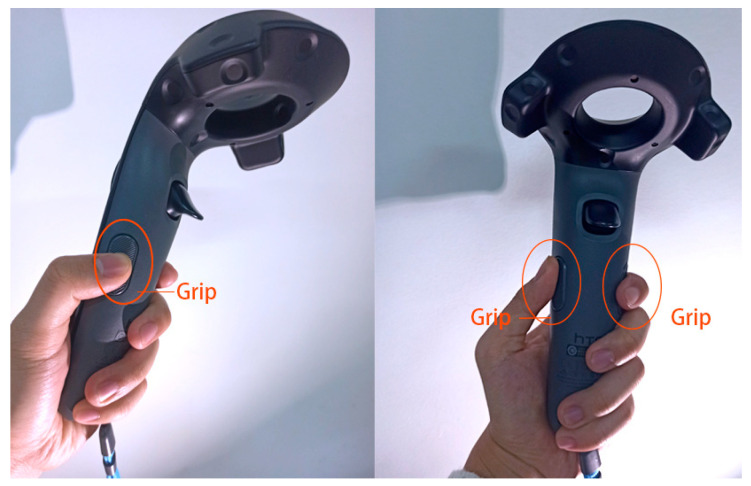
Using the grip key of the HTC Vive handle as the climbing grip key.

We purposefully designed chest expansion exercises and muscle stretching in the game. To overcome specified challenges, participants have to perform the specified action; otherwise, failure is likely to present itself. To better fit our design, we set the grip key of the HTC Vive handle instead of the trigger key as the climbing grip key, which increases the difficulty of climbing at the operation level and simulates load movement, as shown in Figure 5.

### 3.3. Software and Hardware

The scenes were developed with Blender and Cinema 4D (C4D). To make the scene valid in VR, VRTK and SteamVR were chosen as the SDK. HTC VIVE, and Unity were chosen as the wearable VR hardware and development platform, respectively.

Briefly, besides the conceptual design and unit tests, the development procedure was as follows:

After validating the VRTK SDK, we constructed the sports scene in Unity. We then associated the VRTK interactable components, the VRTK Climbable Grab Attach, the VRTK Swap Controller Grab Action, and the VRTK Interact Haptics Components to the key objects in the scene. By assigning design features and interactive events to the objects, the scene was discretely upgraded through the development of each unit’s scripts.

## 4. Preliminary Experimental Evaluation

### 4.1. Experimental Configuration

Twenty-five college students were invited to participate. To investigate the influence on physical and mental functionalities, especially upper limb activity and participation rate, achieved by participating in the designed sports activities, in-sports real-time heart rate monitoring, and a post-game evaluation, including observation, interviews, and a questionnaire survey, were conducted.

Considering the pandemic, all participants were chosen from within the campus of the Guangdong University of Technology. Prerequisites that included having no history of epilepsy or other brain diseases, no vision defects, and no 3D vertigo were applied for filtering the participants. The experiment was approved by the school’s ethics committee, and all the participants authorized or signed the consent forms that described the potential adverse effects of the VR sports experience in detail.

**Figure 6:**
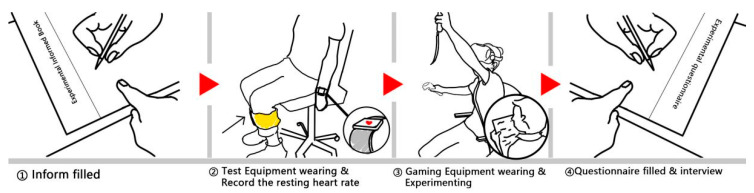
Experimental steps.

Before the tests, the VR Head-Mounted Display (HMD) was adjusted to guarantee sufficient exercise space from the controller and motion sensors. The tests were conducted in a quiet observation room, and each participant was strapped onto a rotatable chair during his/her test. Each test trial lasted for approximately 60 min. Details of the experimental steps are shown in Figure 6.

Firstly, the host introduced the First-Person Sports Game (FPSG) and the test to the participant. In this stage, the test’s purpose, the test procedure, and the precautions were outlined, along with a description of the game’s logic and control. Second, the participant was verbally guided to learn the basic operations and the possible in-game feedback after signing the consent form. Moreover, a heart rate monitor was mounted, and the initial heart rate was recorded as a reference. Third, the participant was instructed on how to wear the VR suite, including the HMD, and handle the remote controller before joining the in-game sports scene. Each trial comprised two sessions: a 10-minute session to get used to the game and a 30-minute session to play the game solo. A non-interfering observer attempted to record the data regarding the participant and the game progress, and the participants’ full-session heart rates were recorded. Lastly, a post-game questionnaire was completed by the participant 3 min after the post-game interview.

### 4.2. Flow Data to Be Collected

In the experiment, a heart rate band was used to measure the participant’s heart rate at rest and during a workout using Photo Plethysmograph (PPG). Through the whole process of recording the participant’s feelings and in the interviews, the in-game psychological effects of the sport on the participants could be evaluated.

According to the theory of flow, five essential dimensions were collected for evaluation. Specifically, data on the sense of control, immersion, satisfaction, feedback, and achievement were collected using five-point Likert scale questionnaires.

After the experiment, we summarized and analyzed the subjects’ feeling state distribution map and used Cronbach’s alpha reliability coefficient to analyze the reliability and consistency of each dimension.

## 5. Results

### 5.1. Basic Statistics

All questionnaires (100%) were successfully collected from the 25 participants. The recruited participants in the experiment had the following characteristics: age: 22.8 ± 1.06; height: 167.48 ± 7.99 cm; heart rate at rest: 78.88 ± 4.96 beats/min. The details of the collected statistics are listed in Table 1.

According to Table 1, most of the subjects could pass the first level; most of the participants had no prior experience of rock-climbing, except for Participants 7, 18, 19, and 20. Most of the participants’ heart rates were significantly elevated after the in-game sport.

### 5.2. User Study of the VR Rock-Climbing Game

According to the results of interviews and observations, the feeling states of the subjects in this study could be summarized into three types of feedback: positive feedback, neutral feedback, and negative feedback, as shown in Table 2. Positive feedback indicates that the subject had a positive psychological response to the experience, including being pleased and excited; neutral feedback indicates that the subject had a neutral psychological response regarding the experience, including considering and being sweaty; negative feedback means that the participant had negative psychological reactions regarding the experience, including being strained and complaining.

**Figure 7:**
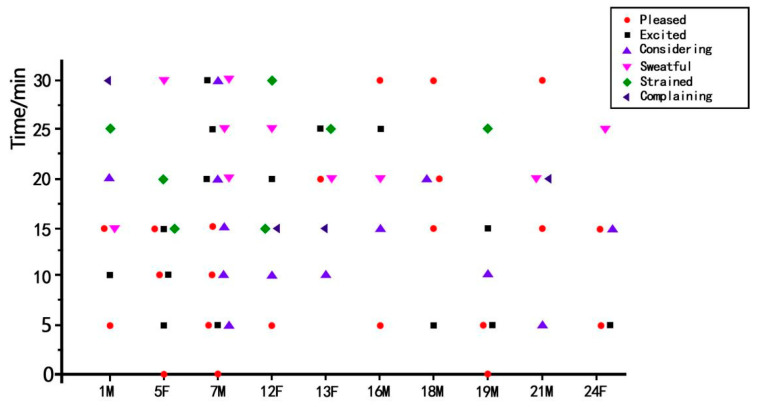
Whole-process feeling distribution of 10 participants.

Subsequently, 10 participants out of 25 were randomly chosen for illustrating the feeling distribution of the whole process, as shown in Figure 7. The abscissa denotes the index and gender (M: male, F: female) of the participants, while the ordinate denotes the duration.

According to Figure 7, 8 out of 10 participants experienced hyperhidrosis after 15 min. Considering the fact that most participants’ heart rates were elevated after the game, it is inferred that the in-game sports had a positive impact on strengthening cardiopulmonary function. Except for Participant 5, most participants displayed the behavior and reported their experience of pausing to think regarding solutions to overcome the in-game challenges. Except for Participant 13, most participants reported experiencing a state of pleasure and excitement in the early stage of the game, inferring that the in-game sports experience was enjoyable. Moreover, Participants 7, 16, 18, and 21 reported a satisfactory post-game experience, indicating the game was not just about the instant gratification of winning.

To sum up, the participants’ feeling state map shows that the subjects were sweaty, excited, and pleased during the game. The overall heart rate observations showed that the in-game sports potentially promoted the participants’ cardiopulmonary function and indicated a positive impact on the body, both psychologically and emotionally.

Moreover, the strong sense of reality in VR made the participants feel pleased and excited, resulting in a sustainable willingness to participate. Instant gratification is the mechanism to deliver pleasure or fulfillment without delay. Instead of offering instant gratification-oriented games, which are believed to be destructive on the mindset in the long term, the setup of the in-game tasks was challenging, which demanded considerable effort in terms of problem-solving and pathfinding to overcome the difficulties encountered in VR rock-climbing. As expected, it allowed the participants to gain a sense of accomplishment and satisfaction after overcoming difficulties and reaching the assigned destination of rock-climbing.

### 5.3. Flow-Based Questionnaire Analysis

#### 5.3.1. CITC-Based Reliability Enhancement

The results of the reliability analysis are shown in Table A1. Regarding the criteria of the key points defined by Guilford [43] and Taisheng Rong [44], the reliability is considered reliable if Cronbach’s α is greater than 0.7, acceptable if Cronbach’s α is between 0.35 and 0.7, and not reliable if Cronbach’s α is lower than 0.35. According to the corrected item–total correlation (CITC) procedure [45], we filtered out results with CITC < 0.4 to improve the reliability of the results, thus enhancing the Cronbach’s α of the collected data. Specifically, an item was removed if both of the following were satisfied: CITC < 0.4 and Cronbach’s α increased if the item was removed.

Cronbach’s α of the sense of mastery was 0.763, which means that the questionnaire has good reliability. According to Table A1 (See Appendix A), the CITC value of Item 5 was less than 0.4, indicating that it had a weak relationship with the rest of the items. However, this item was retained because Cronbach’s α was not significantly enhanced if the item was removed. The CITC value of Item 18 was less than 0.2, which indicates a weak relationship with the rest of the items. Moreover, Cronbach’s α was higher than 0.763. Thus, the item was removed. After removing this item, Cronbach’s α became 0.822, denoting the higher reliability of the questionnaire regarding the given tasks. Cronbach’s α of satisfaction was 0.895, and the questionnaire was reliable. According to Table A1 (See Appendix A), the CITCs of all items were higher than 0.4, and they could be retained.

Cronbach’s α of the in-game feedback was 0.210 (α < 0.35), which means that the collected data are not reliable.

Cronbach’s α of the immersion was 0.617 (0.35 < α <0.7), and the questionnaire was thus considered to be acceptable. According to Table A1 (See Appendix A), the CITC value of Item 20 is less than 0.2, indicating that it has a weak relationship with the rest of the items. The item was removed since the Cronbach α of 0.669, after its removal, was greater than the original value of 0.617.

Cronbach’s α of the sense of achievement was 0.613 (0.35 < α <0.7), and the reliability of the questionnaire was considered to be acceptable. According to Table A1 (See Appendix A), the CITC of Item 26 is less than 0.4, indicating that it is not closely correlated with the rest of the collected items. Item 26 was removed since the Cronbach α significantly increased to 0.739.

According to the CITC and Cronbach’s α-based trimming, after removing Items 18, 20, and 26, the reliability of the collected questionnaire was enhanced for further analysis.

#### 5.3.2. Analysis of Flow Experience

According to Table 3, the average value of each dimension is greater than 4, while the standard deviation is between 0.76 and 0.91, denoting a satisfactory overall in-game experience. Specifically, the average score of the participants’ experience in the control dimension is 4.011, which is greater than 4, and the SD is 0.894, indicating that most participants had a better sense of mastery in the VR rock-climbing game. The participants had clear goals, could master the skills required for operation, and could control the roles. The behavior and operation were consistent, the execution was smooth, and the game’s operation had a high degree of mastery. The average score of the satisfaction dimension is 4.145, which is greater than 4, and the SD is 0.870, indicating that most of the participants were very satisfied with the VR game and found it worthwhile. Additionally, they experienced the excitement of sports during participation, while positive signs of recommendation intentions were reported so that they could explore further, both in the game and in this type of participation.

Note that the average immersion score is 4.336, which is greater than 4, with an SD of 0.769. This result means that most of the participants were immersed in the game and expected to act as egocentric characters in the scene; the average score of accomplishment is 4.020, which is greater than 4, with an SD of 0.905, indicating that most participants acquired a remarkable sense of accomplishment in the VR game. Moreover, their positive experience also implies confidence in overcoming the difficulties in the game.

The collected flow data show that the VR sports game successfully helped participants to be involved and entertained. In the virtual scene, the participants experienced an enjoyable journey of task-oriented sports practice, along with a strong sense of immersion, although the lower parts of their bodies were not involved. As reported, triggered by task-oriented practice and promoted by events in the game, participants reported a strong positive sense of achievement and accomplishment. As willingness to further explore or experience the VR scene was observed, it can be suggested that practice of this kind is promising for increasing the exercise experience and duration of youth with lower-limb dysfunction while restricting the possibility of accidental risk in the outdoors.

### 5.4. Feedback from Participants

According to the post-game interview evaluation, there were five aspects to be improved. First, the visual impact and element richness in the VR scene was ordinary. Second, a couple of troubles with interaction were observed, such as a lag in the warning prompt for the wrong operation, the auditory feedback signals interfering with each other rather than being well distinguished, a long-lasting cognitive burden regarding feedback, and the ambiguity of real-time hints and instructive clues. Third, some troubles in operation were observed, such as pressing a button by mistake, finger tiredness caused by grasping, and the learning burden of becoming used to the scene. Fourth, the participants reported a strong feeling of frustration after failures. Fifth, they reported a lack of hierarchical and progressive gratification, such as overvalue in the immediate gratification, a lack of surrealistic feedback in the game, and expectations of a fascinating narrative story accompanying the gaming progress.

## 6. Discussion

The exercise experience has provided profound insights into gamification design and development:

First, although most participants reported a workout-like experience, they believed that the exercise was more like a generalized exercise rather than real rock-climbing. In other words, the use of muscle groups and exercise intensity were different from those of real rock-climbing.

Second, although the participants commonly reported an enjoyable experience, they agreed that the positive experience came more from the surreal game-play and the pleasure brought by physical body exercise rather than the rock-climbing itself.

Third, the user’s visual experience was like being in a rock-climbing scenario, but the physical perception dominated by haptic feedback was very different from that of rock-climbing. In fact, participants only needed to move the VR handle to the designated position around the body in space during the in-game climbing process. In contrast, the upper limb force of resisting gravity and the activity of keeping the body balanced during real rock-climbing was not well simulated.

In recent decades, virtual reality has been widely adopted as a promising technology to provide feasible, affordable, and customized health promotion and physical rehabilitation solutions. Besides the discussion on commercial interests and the downsides of addiction, the profound social impact on people facing psychological or physical challenges in social life is gaining more attention from the relevant policymakers, rehabilitation specialists, researchers, and developers. The virtual reality solutions emphasize the construction of simulated scenes parallel to the real world perceived by human-embodied cognitive capabilities.

The fact is that virtual reality simulates designed scenes visually. It is still not easy to reproduce the physical laws and feedback fully induced by human perceptual organs in the corresponding real world. For example, although the screen-based digital presentation can simulate visual experiences in movements, its embodied perception and interactive feeling cannot claim to be identical to that in the real world. The former can bring vital psychological comfort to participants and make them happy about being within the sports scene. At the same time, the latter may lead to significant differences between the behavior of participants in virtual reality and those in real-world practice.

There have been theoretical, laboratory, and clinical attempts and practices in societies to extend the sensory dimension and quality of presence and interaction experience in VR gamification rehabilitation. However, such innovations with unique strengths always come with extra costs. Dedicated hardware or specified actuators can vividly simulate haptic feedback in the virtual environment [46], while the high price leads to foreseeable limited applicable scenarios, especially for families.

Alternatively, [47] enforced “flexible” visual augmentations to reinforce “presence”. [48] took advantage of an extrasensory perception over the five primary senses, a hybrid surreal and augmented surrounding which promotes “immersion”.

However, including these mentioned above, few state-of-art works have considered user-centric design usability and feasibility the top essential goals. Our design, an entertaining and cognitively burden-free gamification practice, carefully balances mechanics, space, rules, and components with the goals of enjoyable rehabilitation. It aims to stimulate health promotion-oriented exercise rather than reconstruct an embodied cognitive VR experience identical to real rock-climbing. Moreover, we innovatively integrated the on-purpose design in-game, which is expected to train the aimed muscle group.

According to the pilot study and the psychological flow data collected, the VR software can motivate the intended users by providing artificial VR scenes with surreal enjoyable tasks that are logically compact, with a sense of reward and achievement after overcoming challenges. Although users’ behavior and physical perceptions in virtual reality sports differ from rock-climbing in the real world, it stimulates the upper limb muscles through task-oriented exercise, achieving the ideal effect of sports-based health promotion and rehabilitation.

## 7. Conclusions

In this study, we designed and developed interactive VR rock-climbing software to help people with lower-limb dysfunction participate in immersive and enjoyable upper body exercise. The software allows health-promotion-oriented enjoyable exercise for their upper body, especially the arm, wrist, and pectoral muscle groups. As an in-house leisure and exercise solution, it avoids the social panic and potential risks of a public exercise center or the outdoors. According to the post-game survey, the developed VR solution triggered an immersive, satisfactory, and workout-like overall experience.

Our work differs from relevant works in the following aspects: First, we designed and developed a task-oriented VR sports hybrid solution for adolescents with lower-limb disability towards home entertainment and convenient rehabilitation exercise. Second, we put effort into design usability and feasibility aspects that are socio-technically significant rather than pure technical consideration. Besides user-centric design, the gamification strategy and on-purpose design lead to a particular course of treatment for targeted muscle groups. Third, we conducted real-person evaluations following the flow, which confirmed the designed solution’s mastery, satisfaction, immersion, and achievements. At the same time, the limitation of this work is also notable. The flow evaluation cannot sufficiently assess the task-oriented on-purpose design and lacks the long-term effects on the participants. Moreover, although positive feedback on exercise and rehabilitation is observed, the impacts of our design on the rehabilitation of adolescents with lower-limb disabilities have not been rigorously evaluated by rehabilitation medical experts in clinical experiments. At the same time, the participating imitators may behave differently with adolescents with fixed lower limbs.

In future work, we aim to improve the VR solution to better simulate rock-climbing in terms of the embodied cognitive aspects. Additional physical interactive hardware or accessories are essential for providing realistic somatosensory feedback. Considering the applicability, usability, and feasibility of the design, a non-trivial trade-off can be expected. Moreover, we also plan to invite participants with low limb dysfunctions to join the study since their sports capability and behavior habits are different from those of the participating imitators. Finally, we plan to conduct a study to investigate the attitudes of the family members of the participants to the VR exercise solution with different surreal and game attraction levels as VR sports game addiction is also a concern of family members since the participants temporarily escape from reality while gaming. A fatigue warning could be triggered if the game proves to be over-addictive.

## Figures and Tables

**Table 1:** Statistics of the participants.

Participants	Gender	Age	BMI	Task Clearance (Level and Sublevel)	Heart Rate before Game (per min)	Heart Rate after Game (per min)	Rock-Climb Experience (Yes/No)
1	male	23	20.8	1-5	74	115	no
2	male	22	20.4	2-1	82	99	no
3	male	23	22.1	2-1	93	101	no
4	female	24	21.7	2-1	76	104	no
5	female	23	22.0	2-1	81	92	no
6	male	24	21.6	1-1	83	93	no
7	male	22	23.2	1-6	73	88	yes
8	female	23	21.5	1-2	88	98	no
9	female	24	20.8	1-5	85	99	no
10	male	23	21.4	2-3	70	83	no
11	male	21	20.3	2-1	81	104	no
12	female	22	21.1	1-1	76	96	no
13	female	22	22.9	2-3	75	89	no
14	male	23	20.7	2-1	74	89	no
15	female	25	22.2	2-1	80	100	no
16	male	23	21.2	2-1	75	90	no
17	female	24	21.6	2-1	74	91	no
18	male	22	21.8	1-6	83	94	yes
19	male	22	20.5	2-3	74	101	yes
20	female	22	21.2	1-5	88	96	yes
21	male	24	21.3	2-3	81	90	no
22	male	21	22.1	1-5	75	89	no
23	male	23	21.1	1-1	81	93	no
24	female	24	23.1	2-1	74	99	no
25	male	21	20.7	1-6	76	106	no

**Table 2:** Participants’ feeling states.

Positive Feedback	Neutral Feedback	Negative Feedback
pleased	considering	strained
excited	sweaty	complaining

**Table 3:** Experience score statistics of the dimensions after trimming.

	Cronbach α	Mean	SD	Min	Max
sense of mastery	0.822	4.011	0.894	1	5
sense of satisfaction	0.895	4.145	0.870	1	5
sense of immersion	0.669	4.336	0.769	2	5
sense of achievement	0.739	4.020	0.905	1	5

## Data Availability

Data sharing is not applicable.

## Supplementary Materials

The following supporting information can be downloaded at: https://www.mdpi.com/article/10.3390/ijerph19073985/s1, Video S1: Design to Assist Better Youthhood for Adolescents with Lower Limb Disability through Virtual Reality Sports.

## Appendix A. Supplementary Data

**Table A1:** Items and reliability metric values of different dimensions.

Dimensions	Item	CITC	Cronbach’s α If Item Was Removed
sense of mastery	1. clear understanding regarding tasks and goals	0.605	0.721
	2. mastering the skills needed in the game	0.634	0.702
	5. clear understanding regarding subtasks and subgoals	0.227	0.777
	6. controlling the character’s behavior at will	0.663	0.696
	8. good match between tasks and my capability in controlling the character’s actions	0.437	0.742
	10. operation in the game is coherent and executed smoothly	0.681	0.694
	18. the sense of challenge	−0.067	0.822
	19. proficiency in game operation	0.626	0.707
sense of satisfaction	3. the game is fun	0.689	0.883
	12. the game excites me	0.638	0.889
	15. in-game feeling A (uninteresting–interesting)	0.573	0.889
	16. in-game feeling B (boredom–excitement)	0.644	0.885
	17. in-game feeling C (unhappy–happy)	0.641	0.886
	21. appreciation throughout the game	0.484	0.893
	22. willing to play this type of game again	0.703	0.881
	23. satisfaction with the game’s outcome	0.573	0.889
	25. willing to recommend it to friends or relatives	0.530	0.891
	27. inspired to explore more ways of climbing	0.756	0.878
	28. feeling the pleasure of sports	0.709	0.881
sense of immersion	7. high concentration on the game, ignoring the surrounding of the real world	0.441	0.533
	9. high concentration on the game, losing track of time	0.462	0.546
	13. feeling like being in another world while in the game	0.475	0.515
	14. in-game peace dominates	0.500	0.534
	20. deep aftertaste after game	0.111	0.669
	24. virtual in-game reality replaces reality	0.245	0.621
sense of achievement	26. knowledge learnt from the game	0.248	0.739
	29. sense of milestones achieved in-game	0.387	0.561
	30. confidence to overcome difficulties in-game	0.679	0.073
